# Functional genomics of acclimation and adaptation in response to thermal stress in *Daphnia*

**DOI:** 10.1186/1471-2164-15-859

**Published:** 2014-10-04

**Authors:** Lev Y Yampolsky, Erliang Zeng, Jacqueline Lopez, Patricia J Williams, Kenneth B Dick, John K Colbourne, Michael E Pfrender

**Affiliations:** Department of Biological Sciences, East Tennessee State University, Johnson City, TN 37641 USA; Department of Computer Science and Engineering, University of Notre Dame, Notre Dame, IN 46556 USA; Department of Biology and Department of Computer Science, University of South Dakota, Vermillion, SD 57069 USA; Department of Biological Sciences and Environmental Change Initiative, University of Notre Dame, Notre Dame, IN 46556 USA; School of Natural Sciences Lenoir-Rhyne University, Hickory, NC 28603 USA; Environmental Genomics Group, School of Biosciences, University of Birmingham, Birmingham, UK

**Keywords:** Gene expression, Thermal tolerance, Temperature, Plasticity, GxE, Metabolic compensation, Canalization

## Abstract

**Background:**

Gene expression regulation is one of the fundamental mechanisms of phenotypic plasticity and is expected to respond to selection in conditions favoring phenotypic response. The observation that many organisms increase their stress tolerance after acclimation to moderate levels of stress is an example of plasticity which has been long hypothesized to be based on adaptive changes in gene expression. We report genome-wide patterns of gene expression in two heat-tolerant and two heat-sensitive parthenogenetic clones of the zooplankton crustacean *Daphnia pulex* exposed for three generations to either optimal (18°C) or substressful (28°C) temperature.

**Results:**

A large number of genes responded to temperature and many demonstrated a significant genotype-by-environment (GxE) interaction. Among genes with a significant GxE there were approximately equally frequent instances of canalization, i.e. stronger plasticity in heat-sensitive than in heat-tolerant clones, and of enhancement of plasticity along the evolutionary vector toward heat tolerance. The strongest response observed is the across-the-board down-regulation of a variety of genes occurring in heat-tolerant, but not in heat-sensitive clones. This response is particularly obvious among genes involved in core metabolic pathways and those responsible for transcription, translation and DNA repair.

**Conclusions:**

The observed down-regulation of metabolism, consistent with previous findings in yeast and *Drosophila*, may reflect a general compensatory stress response. The associated down-regulation of DNA repair pathways potentially creates a trade-off between short-term benefits of survival at high temperature and long-term costs of accelerated mutation accumulation.

**Electronic supplementary material:**

The online version of this article (doi:10.1186/1471-2164-15-859) contains supplementary material, which is available to authorized users.

## Background

Organisms respond to environmental changes by adjusting their physiology, biochemistry, behavior, and sometimes, morphology. The ability of a single genotype to generate a variety of phenotypes in response to environmental changes is termed phenotypic plasticity and the resulting increase of tolerance to stressful levels of environmental parameters is known as acclimation. One of the central goals in the study of adaptive phenotypic plasticity has been the analysis of reaction norms in ancestral and evolved populations [1–3]. In the postgenomic era such analysis is a necessary condition for answering one of the major emerging questions in evolutionary and ecological genomics: are the genes involved in plastic responses the same as those underlying adaptive differentiation [4]? Using the emerging model organism *Daphnia,* we address this question by analyzing the differential expression patterns of heat-tolerant and heat-sensitive genotypes that have been acclimated to either optimal or stressfully high temperature.

One fundamental molecular mechanism of phenotypic plasticity acclimation is up- or down-regulation of the expression of individual genes to meet the organism’s needs prescribed by the changing environment [5, 6]. Phenotypic plasticity is easy to observe but difficult to interpret. In particular, it can be difficult to untangle plasticity and adaptation, as adaptation may be achieved by the evolution of plasticity rather than by the evolution of a constitutive tolerance mechanism.

A further complication is that it may be difficult to demonstrate that phenotypic plasticity at the level of gene regulation is causative of higher fitness in the inducing environment. Correlated patterns of expression due to regulatory or developmental constraints [1, 7] may cause cascades of genes to respond in a concordant manner, seemingly to match the environmental demand. For example, among the large number of genes responding to early exposure to ethanol in *Drosophila* embryos [8] there are genes that are likely downstream responses to ethanol presence and thus non-adaptive in the sense of increased ethanol tolerance. Similarly, if genes respond to the same transcriptional regulators with the causative, adaptively plastic genes, the correlated response will make it difficult to single out the causative genes. In cases where a small number of candidate genes are implicated in adaptive plastic responses it is possible to employ direct gene-specific manipulative fitness-measuring approaches. However, when a significant portion of the genome responds to a specific environmental cue testing the fitness effect of each instance of differential expression becomes impractical. Validating the relationship between regulatory plasticity and adaptive value is particularly difficult in non-model systems lacking well-established reverse genetic approaches. In addition, the lack of functional annotations for many genes in non-model species complicates establishing the relationship between genes and phenotypes.

One plausible approach is the measurement of gene expression rates in a common-garden experiment in which stress-tolerant and stress-sensitive genotypes are exposed to the same level of stress. While this approach is still subject to the difficulties caused by correlated responses it allows the identification of groups of co-regulated genes whose regulation is likely to be adaptive. Expression responses to the environment observed in stress-tolerant, but not stress-sensitive genotypes, are likely to represent an evolved mechanism rather than be a product of a regulatory constraint. Likewise, genes that are plastic in sensitive genotypes, but constitutive in tolerant ones, are likely to demonstrate adaptive elimination of misadaptive environmental response, i.e., canalization. Surprisingly, such common-garden experiments are scarce both in model [8–10] and non-model organisms [11, 12]. To the best of our knowledge, here we report the first study of transcriptome response to temperature in *Daphnia*, a classic model for studies of phenotypic plasticity, and the first such study in the context of heat tolerance.

Facing stressful environmental conditions an organism can respond either by minimizing damage, by reducing demand/increasing efficiency of the consumption of resources related to stress-tolerance, or by escaping the stressful environments. Investigations of the plasticity of heat tolerance in aquatic ectotherms have provided examples of all these response mechanisms. For example, thermal damage can be minimized by the chaperoning activity of heat shock proteins [13] or by activation of P450 cytochromes, recently implicated in a variety of heat-tolerance responses, possibly through their involvement in oxidative stress metabolic pathways [14, 15]. Organisms in stressful low or high temperature environments can also implement membrane restructuring to achieve sufficient membrane fluidity at low temperatures without sacrificing structural stability, or suffering too much ion leakage at high temperatures [16, 17]. Transcriptional responses of some of these specific heat response genes will be reported elsewhere (in preparation); here we will focus on genome-wide patterns of response.

In aquatic organisms high-temperature tolerance is often constrained by the mismatch between metabolic oxygen demand and oxygen availability. Indeed, metabolic demands typically increase exponentially with temperature, while oxygen availability is reduced by the lower solubility of oxygen in water at higher temperatures [18–21]. Numerous aquatic ectotherms are known to respond to elevated temperature by over expressing haemoglobins, which allows them to more efficiently transport and store oxygen [21–24]. In *Daphnia* haemoglobins are particularly interesting since they likely play a role in protecting tissues from oxidative damage [21] and are known to be co-expressed with the male-inducing hormonal pathway. The production of males and a transition to sexual reproduction is a possible mechanism to escape unfavorably high temperatures through shifting the reproductive effort to sexually produced diapausing eggs [25].

Another well-documented response of a variety of aquatic organisms to the dilemma posed by metabolic oxygen demand is temperature-induced metabolic compensation [26–28]. This compensatory response is characterized by the across-the-board reduction of metabolic activity at temperatures close to the upper tolerance limit. The protein-level mechanisms of this plastic response have attracted a great deal of attention [29], while comparable data on transcriptional response to temperature indicative of the metabolic compensation response are scarce [9, 30].

In this paper we ask the following questions:

1) What is the generalized transcriptome response in *Daphnia* to long-term exposure to near-lethal temperature? 2) Are specific metabolic pathways up- or down-regulated during such acclimation? If so, can these pathways be identified as transcriptional manifestations of known temperature acclimation mechanisms such as metabolic compensation? 3) Are the observed patterns of gene regulation different in a small sample of temperature-sensitive vs. temperature-resistant genotypes? and 4) For genes showing a significant genotype-by-environment interaction, is the magnitude of differential expression in response to temperature greater or less in heat-tolerant or heat-sensitive genotypes? If differential expression is adaptive, and if local adaptation occurs through evolution of plasticity, we expect to observe an increase in plasticity in heat-tolerant compared to heat-sensitive genotypes. An adaptive expansion of plasticity is often described as the initial phase of the Baldwin effect [31, 32]. Conversely, greater plasticity in heat-sensitive compared to heat-tolerant genotypes would be indicative of canalization playing a role in adaptive changes in gene regulation.

To summarize, we are reporting a genome-wide analysis of evolutionary patterns of transcriptional response during acclimation to high temperature in *Daphnia*, an emerging model system for ecological genomics.

## Results

### Methodology overview

We analyzed the transcriptional profiles of four *Daphnia* genotypes, previously classified as either “heat tolerant” or “heat sensitive”, at two temperatures using microarray technology. For each gene the difference in mean expression between the two temperatures is a proxy for the phenotypic plasticity of this gene’s expression. The difference in the mean expression levels observed in heat tolerant and heat sensitive genotypes is, with reservations, a proxy for the constitutive adaptation of a gene’s expression level. Expression values were obtained for 29212 protein-coding genes annotated in the *Daphnia pulex* genome. No filtering for genes with expression level significantly higher than the background was employed to keep multiple test corrections conservative. A False Discovery Rate (FDR [33]) cut-off of 0.05 was used throughout the analysis. Functional gene annotations, including memberships in clusters of eukaryotic orthologous groups (KOGs [34]; see Methods for details), a widely used orthology-based functional classification were obtained from the *Daphnia* genome database (wfleabase.org).

### Overall response to temperature

**Figure 1:**
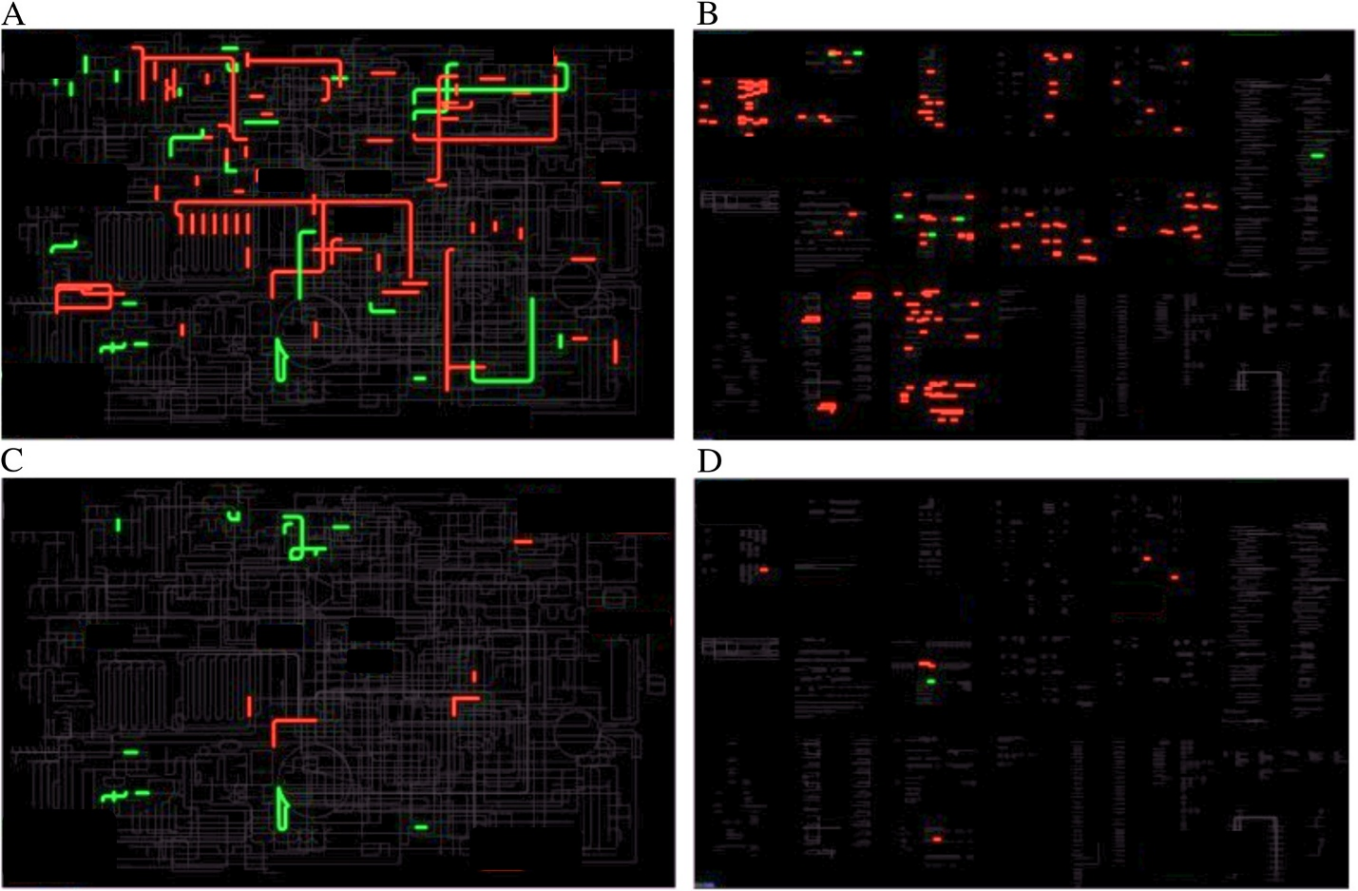
**iPATH representation of differentially expressed genes in either Southern (A,B) or Northern (C,D) genotypes. A**, **C**: metabolic pathways. **B**, **D**: regulatory pathways. Compare to Figure 2 for annotations of major pathways.

The predominant genome-wide transcriptional responses occurring after 3 generations of acclimation to 28°C were the down-regulation of significant portions of metabolic and regulatory pathways. When all four clones are considered together, 892 KOGs were found to contain significantly down-regulated genes, while only 295 KOGs contained up-regulated genes. The same numbers for individual genes were 1549 genes down-regulated and 907 genes up-regulated. When northern and southern clones are considered separately (Table 1, Figure 1), the number of significantly up- and down-regulated pathways and individual genes were more similar, but still, down-regulated genes outnumbered the up-regulated ones.

**Figure 2:**
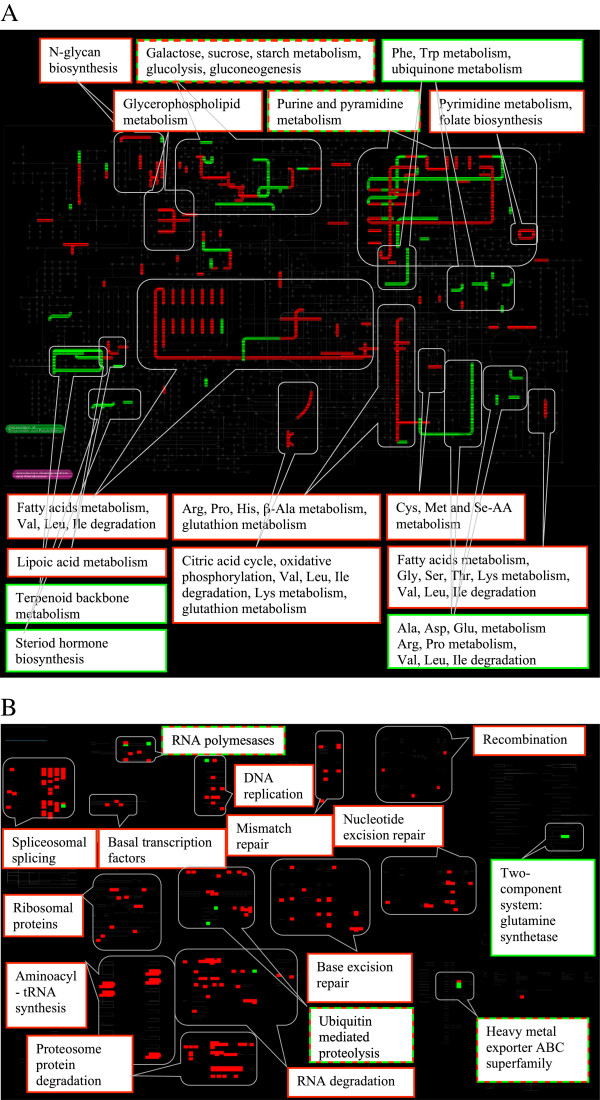
**Annotated iPATH representation of differentially expressed genes (significant temperature effect in all data). A**: metabolic pathways; **B**: regulatory pathways. Red: down-regulation at 28°C relative to 18°C, Green: up-regulation.

Numerous down-regulated KOGs included proteins participating in fatty acid and amino acid metabolism pathways (Figure 2A). There were three standouts among the largely down-regulated lipid metabolism pathways steps (green elements in Figure 2A). One such standout was the step leading to palmitoil-coA, which contains an up-regulated enzyme described as very-long-chain specific acyl-coA dehydrogenase, possibly indicating a shift in the spectrum of lipids produced. Two other up-regulated lipid metabolism pathways were the steroid hormones biosynthesis pathway and terpenoid (steroid hormone precursor) metabolism pathway.

**Table 1:** **Numbers of differentially expressed (FDR < 0.05) genes in Northern (N, sensitive) and Southern (S, tolerant) genotypes (ns = non-significant)**

	N, ns	N, up	N, down	Total
S, ns	27315	54	113	27482
S, up	719	46	0	765
S, down	942	0	23	965
Total	28976	100	136	29212

For example, 46 genes were found to be up-regulated in both N and S populations.

Similarly, although many steps of some amino acid metabolism were down-regulated, some were up-regulated (Ala, Asp, Glu, Arg, Trp and Pro) and some parts of Val, Leu and Ile degradation pathways were up-, while others were down-regulated. The nucleotide metabolism pathway showed a patchwork of up- and down-regulated steps.

A clear pattern emerged for regulatory pathways gene expression (Figure 1B, D). The experimental group acclimated to 28°C showed across the board down-regulation of nearly all KOGs participating in DNA replication (except Ribonuclease H, which is up-regulated), transcription (except the large subunit of RNA polymerase II and the subunit E), splicing (except splicing factors from RRM and SR superfamilies) and translation, including both the expression of ribosomal proteins and aminoacyl-tRNA synthetases. Interestingly, the aminoacyl-tRNA synthetases that showed a significant down-regulation (specifically, alanyl-, asparagyl-, leucyl-, isoleucyl- and tryptophanyl-tRNA synthetases) serve the amino acids that showed either up-regulated metabolism or a down-regulated degradation pathway (Figure 2A). Degradation pathways that were down-regulated included ubiquitin-mediated proteolysis (except E3 ubiquitin ligase, which was up-regulated), proteosome protein degradation and RNA degradation (except 5′-3′ exonuclease HKE1/RAT1, which was up-regulated). Another pathway showing a mixed up- and down regulatory pattern was the ABC superfamily of heavy metal exporter proteins. Uniformly down-regulated genes included genes participating in homologous recombination as well as mismatch, nucleotide excision and base excision DNA repair.

The fact that some pathways were highlighted on the iPATH map while others were not is not necessarily indicative of significant up- or down-regulation of the entire pathways or overrepresentation of members of such pathways among differentially expressed genes. It may rather be a manifestation of the large number of different KOGs participating in a given pathway. None of the pathways highlighted on Figure 2 (out of the total of 125 metabolic pathways) were significantly (after FDR correction) enriched in differentially expressed genes as indicated by Fisher’s Exact Test (data not reported). Thus, the comparison between highlighted and non-highlighted pathways is not meaningful. However, meaningful comparisons are between pathways that contain up- vs. down-regulated elements, and between pathways whose elements are differentially expressed in some, but not other genotypes.

Indeed, the observed patterns were drastically different when the two heat-tolerant southern genotypes and the two heat-sensitive northern genotypes are considered separately (Figure 1). While some parts of metabolism were regulated in these two types of clones similarly (e.g., fatty acid metabolism), others were not. The complex up- and down-regulation of nucleotide metabolism was seen in heat-tolerant, but not in heat-sensitive genoptypes, as were the down-regulation of N-glycan synthesis and the up-regulation of steroid hormone biosynthesis pathway. Several steps of ubiquinone and other terpenoid-quinone biosynthesis were up-regulated in heat-tolerant, but not in heat-sensitive genotypes (Figure 1A,C). Intriguingly, retinol metabolism was up-regulated in heat-tolerant, but (non-significantly) down-regulated in heat-sensitive genotypes.

Finally, a discrepancy was observed between the analysis of terpenoid backbone biosynthesis pathway using both Northern and Southern genotypes in the complete model (Figure 2A) and the patterns that were observed in Northern and Southern genotypes considered separately. The complete model indicated that this pathway contained an up-regulated enzyme, trans-pentaprenyl transferase (EC:2.5.1.33), which was not significantly up- or down-regulated in either the Northern or Southern genotypes analyzed separately. In contrast, geranyl pyrophosphate synthase, an enzyme catalyzing a neighboring pathway step, was significantly down-regulated in Southern, but not the Northern genotypes (Figure 1A,C).

An even more striking pattern of differences between Northern and Southern genotypes was observed in the gene expression of regulatory genes. Many more regulatory genes were down-regulated in heat-resistant than in heat-sensitive genotypes (Figure 1C,D). Heat-sensitive genotypes completely lack the characteristic down-regulation of DNA replication and repair genes, and genes responsible for transcription and splicing observed in heat-tolerant genotypes. If this observation is correct, it indicates that acclimation to stressful subleathal elevated temperatures in *Daphnia* may be accompanied by systemic down-regulation of DNA replication and gene expression machineries and that this pattern is associated with the increased tolerance to thermal stress found in the Southern genotypes.

### Canalization vs. enhancement of plasticity in heat adaptation

**Figure 3:**
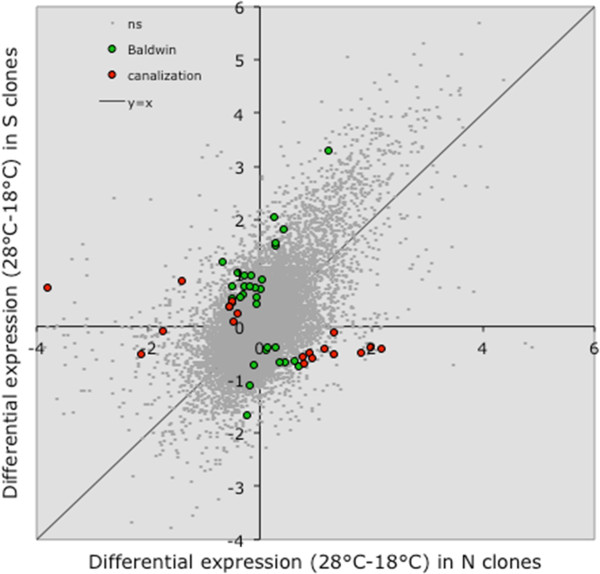
Genes with significant T*geo interaction (tested against the pooled random MS; FDR q < 0.05), for which the Southern (heat-tolerant) genotypes show greater absolute difference in expression at two temperatures (green symbols, Baldwin effect) or smaller absolute difference in expression at the two temperatures (red symbols, canalization) than the Northern (heat sensitive) genotypes.

The presence of genotype-by-environment interaction in the comparison of geographically distinct genotypes may indicate the evolution of plasticity, including plasticity of gene expression. Assuming that heat-sensitive Northern genotypes represent the ancestral state and the heat-tolerant Southern genotypes represent the derived state, one may test whether this local differentiation occurred through genetic canalization (i.e., a reduction of plasticity where the inducible phenotype becomes constitutive) or an expansion of plasticity (i.e., Baldwin effect). Although the results in Figure 1 and Table 1 might indicate a widespread Baldwin effect (tolerant genotypes show differential expression, while sensitive clones do not), in reality there are nearly as many cases among genes with a significant (FDR q < 0.05) T*geo effect in which the Southern (heat tolerant) genotypes show a greater plasticity of expression than the Northern (sensitive) ones (Figure 3).

### Genome-wide principal component analysis

**Figure 4:**
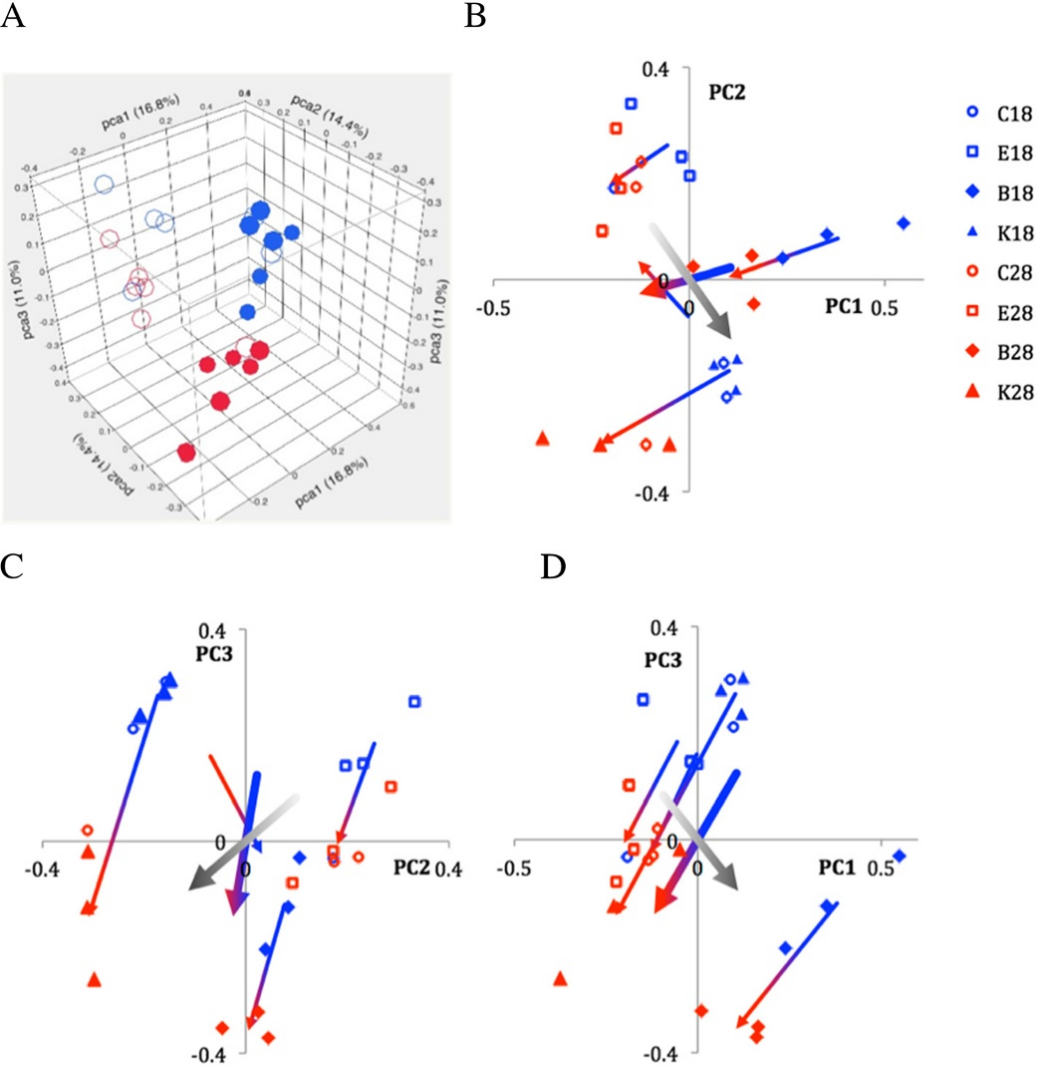
**Principal components analysis of differential expression of 29,212 genes represented on the array in 4 clones of** ***Daphnia*** **from Northern (open symbols) and Southern (solid symbols) acclimated to either 18°C (blue symbols) or 28°C (red symbols). A**: RNA samples in the 3D space of the first three principal components, together explaining 42.2% of variance. **B** – **D**: the same on pairwise 2D planes of the first three principal components. Arrows connect the Euclidean centroids of 3 replicates for each clone at each temperature (thin blue-red gradient arrows) or the centroids of all replicates at each of the two temperatures (thick blue-red gradient arrows) or at each of the two geographic origins (N vs. S, white-black gradient arrows).

Figure 4 represents the principal component analysis of RNA samples in the space of transcript abundance values of the 29,212 genes represented on the array. There is a clear separation between the Northern and Southern samples (open and solid symbols) and well as between the samples obtained from *Daphnia* acclimated to 18°C vs. 28°C (blue and red symbols; Figure 4A). A more detailed representation on pair-wise planes of the first three principal components (Figure 4B-D) shows a clear separation of biological replicates corresponding to each of the four clones. One exception is the Northern genotype C with three replicates (2 in 28°C and one in 18°C) located far from the other three replicates of this genotype and within the cloud of the Southern genotypes.

## Discussion

We analyzed the genome-wide transcription profiles of two Northern, heat-sensitive and two Southern, heat-tolerant genotypes of *D. pulex* that were acclimated to either 18°C or 28°C*.* We observed a widespread down-regulation of expression in a variety of metabolic and regulatory pathways. This pattern of down-regulation was more pronounced in heat-tolerant genotypes. Because stressfully-high temperatures in aquatic ectotherms exacerbate the discordance between metabolic oxygen demand and oxygen solubility [18–20], we hypothesize that the observed down-regulation may be the molecular mechanism of metabolic compensatory reaction [28] to nearly lethal temperature. This finding is consistent with the growing body of evidence from yeast [30] and *Drosophila* [9] indicting that high temperature (as well as other stressors) can elicit genome-wide down-regulatory responses. The fact that this reaction is observed in heat-tolerant, but not in heat-sensitive genotypes suggests that it may be the causative mechanism of heat tolerance and not a direct non-adaptive effect of high temperature. This finding is in a striking disagreement with the results of a recent study on *Drosophila* suggesting that tropical (i.e., adapted) populations are more likely to exhibit up-regulation of genes at 30°C than temperate populations [9]. Levine et al. [9] argued that this result suggests higher genome-wide levels of gene expression at temperatures most commonly experienced in the region of origin. The difference between our study and Levine et al. [9] may reflect real biological and ecological differences between the study organisms. Perhaps since *Drosophila* can escape overheating by behavioral thermal regulation they may show a different transcriptional response to stressful elevated temperature than do *Daphnia* from small ponds. The aquatic thermal environment is variable through time, but at any particular time point it is homogeneous compared to the terrestrial environment

How confident can we be that the observed differences between the two Northern, heat-sensitive and the two Southern, heat-tolerant genotypes are indeed functionally related to the evolutionary history of temperature adaptation in these spatially distinct populations? Certainly a larger sample size (feasible with RNAseq approaches, but logistically difficult within the microarray approach employed here) could have provided stronger evidence. There are two arguments that provide some support for the conclusion that the observed patterns are not independent from the evolutionary history of the populations used in this study. First, local populations of *D. pulex* in North America have long been known to show a high degree of populations subdivision, swamping the north–south differentiation [35]. This observation suggests that it is not likely that the two Southern or the two Northern genotypes, each derived from different local populations, have a recent common origin. Thus, the potentially confounding factor of phylogenetic relationship is likely minimal and our sample size is two per geographic region. Second, heat tolerance has been shown to be highly correlated with the climatic conditions and/or latitude, using a larger number of clones, both in N. American *D. pulex* [36] and on world-wide sample in a congeneric *D. magna* [37]. Still, given the low number of replicates, the geographic patterns described here should be treated as a suggestion, not a solid proof of local adaptation to thermal environment.

The fact that DNA repair pathways appeared to be down-regulated, opens an intriguing possibility of a mutagenic effect of high temperatures in those genotypes that demonstrate such down-regulation. The rate of spontaneous mutation is likely to be higher at elevated temperatures for thermodynamic reasons and the down-regulation of DNA repair should exacerbate this effect. As a consequence genotypes down-regulating DNA repair pathways in response to elevated temperatures may survive periods of high temperature at a cost to a greater mutational pressure.

In a recent study, DNA repair in *Daphnia* has been shown to be much less effective at 20°C than at 10°C [38]. It is, however, not obvious that the down-regulation of DNA repair pathways observed in this study is a manifestation of the same phenomenon. Firstly, in [38] photoreactivation repair of UV damage was measured, while differential expression of photoreactivation pathway has not been detected here. More importantly, the cited study detected the decrease in DNA repair efficiency at the temperature (20°C) that is close to the optimum and far below the substressful temperature at which the down-regulation of DNA repair pathways has been observed in this study.

Several *a priori* expected signals of acclimation to higher temperature are apparent in either all genotypes or in the heat-tolerant genotypes only. These signatures include changes in lipid metabolism potentially indicative of the change in membrane and storage lipid composition – a possibility that warrants a joint transcriptome and lipidome analysis in heat-acclimated *Daphnia*. Another such pathway related to lipid metabolism is the terpenoid-quinone biosynthesis pathway. Unlike many differentially expressed pathways, this pathway was found to be up-regulated when all clones were considered together. Yet, in the separate analysis of Northern and Southern genotypes, this pathway was found to be down-regulated in Northern, but not in Southern genotypes through differential expression of a different enzyme. This observation seems to indicate that during acclimation to high temperature *Daphnia* regulate the terpenoid biosynthesis pathway in a complex way. Both reactions with detected differential expression feed into the sesquiterpenoid biosynthesis pathway leading to methyl farnesoate hormone – a signal molecule known to induce male production [39] as well as regulating the production of haemoglobin in *Daphnia* [25, 40]. How this rearrangement of the terpenoid biosynthesis pathway may fine-tune methyl farnesoate is unknown, but it is quite possible that such a rearrangement may play an important role in temperature dependent haemoglobin expression regulation and the reproductive switch to male production.

A recent study [8] of adaptation and plasticity of gene expression in response to alcohol stress in *Drosophila* reported canalization of gene expression for practically all instances of significant GxE interactions. In contrast, in this study we found approximately equal number of significant T*geo interactions in which the “evolved” (heat-tolerant) genotypes had stronger transcriptional plasticity than the “naïve” (heat-sensitive) genotypes and vise versa. We have previously noted gene specific variation in the pattern of expansion and reduction in the plasticity of gene regulation in the context of pigmentation genes in *Daphnia* [2]. In our current study it appears that temperature-specific differential expression in *Daphnia* may be evolving through both canalization of plasticity and through an adaptive increase of plasticity. It should be noted that it may be hard to detect enhanced plasticity in selected phenotypes because of the ‘Fry conjecture’: selected genotypes are better at handling stress, thus the “internal” stress indicators may not be sufficient to induce expression changes, thus creating a false indication of canalization. This conjecture was proposed by J. Fry to explain lower transcriptional responses to ethanol exposure in ethanol-selected populations than in the controls [8]: ethanol-tolerant genotypes have a higher ethanol-detoxifying ability and thus experience lower internal concentration of ethanol and acetaldehyde, resulting in weaker transcriptional response in other genes. While plausible for xenobiotic stressors (such as alcohol), this mechanism can also work for temperature: certainly heat-tolerant genotypes experience the same temperature, but they may experience a milder level of any downstream parameters (oxygen concentration in tissues, degree of oxidative damage etc.) that may act as transcriptional triggers than the heat-sensitive genotypes. Thus, the question about canalization vs. expansion of plasticity in response to selection will remain not fully resolved until specific trigger mechanisms measurable in ancestral and evolved genotypes can be identified.

In our genome-wide analysis, the samples clearly form four separate clouds in the space of the first three principal components. These four clouds correspond to the two temperatures and two geographic origins (North vs. South), with a few exceptions (Figure 4A). The separation of points corresponding to the two temperatures appears to be stronger in the Southern (tolerant) than in the Northern (sensitive) clones, suggesting overall expanded expression plasticity, i.e., Baldwin effect. One of the genotypes (C) stands out by having three of its biological replicates located in the “wrong” (Southern) cloud. Barring a possibility of mislabeling (unlikely, as only PC2 is affected), this placement may indicate that there are two distinct syndromes, or patterns of gene expression, one typical for the two southern clones and the other typical for the two northern clones, with some clones switching between the two modes haphazardly, with no relation to current temperature. With this single exception, genotype-specific points (biological replicates) cluster together and the arrows connecting 18°C and 28°C centroids are remarkably parallel. In contrast, the summary 18-28°C arrows representing acclimation and the black-and-white arrows representing divergence and possible prior local adaptation (Northern vs. Southern) are not parallel, particularly on the plane of the first two principal components, indicating that selection operating on gene expression in nature may be orthogonal to the plastic response observed during acclimation. One possible explanation of for this is that the plastic genes, due to their plasticity, are shielded from the natural selection operating on the adaptation to elevated temperatures.

## Conclusion

Numerous metabolic and regulatory pathways are regulated during long-term acclimation to high temperature in *D. pulex*. More genes are down-regulated than up-regulated and this effect is stronger in the two Southern, heat tolerant genotypes than in the two Northern, heat sensitive genotypes, suggesting metabolic compensation as a possible acclimation mechanism. Widespread down-regulation of gene expression and DNA repair pathways may represent a “last-resort” survival tactic in organisms facing a trade-off between long-term and short-term survival. Finally, there was a mixture of genes showing reduced plasticity (i.e., canalization) of gene expression in response to temperature and genes that exhibit increased plasticity.

## Methods

### Genotype provenance and acclimation experiment

We chose four genotypes representing two extremes of the heat tolerance gradient measured in [36]: heat tolerant genotypes BW102 (hereafter B) and KSP3 (hereafter K), originated from Illinois and heat sensitive clones EB1 (hereafter E) and CHQ3 (hereafter C), originated from Minnesota and Wisconsin, respectively. These two pairs of genotypes will be hereafter referred to as “Northern” (N) and “Southern” (S) clones. Although the latitudinal difference between N and S clones is only 4–6 degrees, and the difference in average July temperatures is only 2-5°C, there is a significant difference in temperature tolerance between the two pairs of genotypes (Table 2). This difference is likely associated with the microclimate difference between their habitats of origin. Northern ponds are located in wooded areas and are likely to remain cooler during the summer months than the smaller, shallower and more exposed ponds in prairie zone, from which the Southern clones were sampled [36].

**Table 2:** **Genotype origin and characteristics**

Genotype ID	Pond/Lake	State	Lat °N	Long °W	July °T	Performance at 37°C		
						T_imm_	SE	M
BW102	Busey Woods	Illinois	40°07′	88°12′	24.0	2.53	0.053	0.50
KSP3	Kickapond	Illinois	40°06′	88°14′	24.0	2.52	0.054	0.28
CHQ3	Chequamegon	Wisconsin	46°19′	90°54′	19.0	1.89	0.069	0.66
EB1	Eloise Butler	Minnesota	44°59′	93°19′	22.2	1.97	0.046	0.82

Average July temperature: long-term average from the nearest weather station.

Time until immobilization in hours (T_imm_) and mortality (M, loss of heartbeat after 3 hours of exposure) were measured at 37°C in Daphnia acclimauted to 24°C. See [36] for details.

Three replicates of each of the four genotypes were maintained by parthenogenetic reproduction for two generations at either 18°C or 28°C in 150 ml bottles containing COMBO water medium [41] under a 12 L:12D photoperiod. It is worth mentioning that the 28°C treatment is not lethal. While this temperature is detrimental to survival it still allows continuous maintainance and reproduction of organisms allowing for multigenerational acclimation. Food in the form of *Scenedesmus acutus* culture was added daily to the final concentration of 200,000 cells/ml. The medium was replaced and neonates removed twice weekly. To establish the experimental generation, 20–30 second or third clutch offspring of the second-generation females were collected within 48 hours of birth and placed in bottles containing 150 ml of COMBO medium. These third generation individuals were screened after reaching the age of 12–15 days and females with early development stage clutches (round uniformly dark eggs with smooth edges; <24 hours after clutch deposition) were selected for the microarray experiment and stored in liquid nitrogen.

### RNA extraction, reverse transcription, labeling and array hybridization

To assess patterns of gene expression, we used the NimbleGen *D. pulex* Expression Array 12×135k (GEO Accession GPL11278; [42]). Briefly, this platform is a high-density NimbleGen (Roche-NimbleGen, Inc., Madison, WI, USA) gene expression microarray of 12 identical arrays prepared by Maskless Array Synthesizer. Each array contains 137,000 isothermal probes interrogating 35,665 genes. Each predicted and experimentally validated gene is represented by as many as three unique probes, while the remaining probes are designed from transcriptionally active regions (TARs) whose gene models are not yet known.

Third-generation acclimated adult females (10 – 12 individuals) with early stage clutches were homogenized in TRIZOL Reagent (Invitrogen, Carlsbad, CA). The homogenate was purified using Qiagen’s RNeasy Mini Kit (Qiagen,Venlo, Netherlands) with on-column DNAse treatment to isolate total RNA. Beginning with 1.0 μg of total RNA, a single round of amplification using MessageAmpTM II aRNA kit (Ambion, Austin, TX) was performed for each RNA sample. cRNA (10 μg) was converted to double strand cDNA with random primers using the Invitrogen SuperScript Double-Stranded cDNA Synthesis kit (Invitrogen, Carlsbad, CA). From 1 μg double-stranded cDNA, labeled cDNA was generated with NimbleGen’s Dual-colour Labeling Kit (Roche NimbleGen, Inc., Madison, WI). The replicates of each treatment (28°C) and control (18°C) for each geographic origin (Northern and Southern) were alternatively labeled and a dye swap was included among the replicate experiments. Dual-colour hybridization, post-hybridization washing and scanning were done according to the Roche NimbleGen’s instructions. Images were acquired using a GenePix 4200A scanner, 5 μm resolution, and GenePix 6.0 software (Molecular Devices, Sunnyvale, CA). The data from these arrays were extracted using the software NimbleScan 2.4 (Roche NimbleGen, Inc., Madison, WI). Because we were interested in the expression patterns that describe the adaptive differences between the genotypes and the plastic differences within a genotype, we used four competitive hybridizations that define these axes (Northern at 18°C vs. Southern at 18°C; Northern at 28°C vs. Southern at 28°C; Northern at 18°C vs. Northern at 28°C; Southern at 18°C vs. Southern at 28°C) in a loop design where each hybridization was replicated three times with one dye-swap nested in each set of replicates.

### Statistical analysis

The NimbleGen array image data were processed using NimbleScan version 2.5 to extract probe intensity values. Gene expression values (i.e., gene intensity value) were obtained from a summarization of intensity values of all corresponding probes using the RMA (Robust Multi-array Average) method. The pre-processed microarray data were imported into an in-house analysis pipeline using Bioconductor for normalization and analysis [43]. All genes were quantile-normalized across arrays, samples, and replicates [44]. Differential expression was assessed using LIMMA and EBarrays [45, 46] using the median signal of probes representing genes. EBarrays uses a parametric mixture model to calculate the posterior probability of differential expression for arbitrarily complex experimental designs. To determine the significance of expression differences, and adjust for multiple testing, we calculated the False Discovery Rate (FDR; [33] for each gene using the Bioconductor LIMMA package.

The following general linear model was utilized to test for the significance of acclimation temperature (T, 18°C vs.28°C, d.f. = 1), geographic origin (geo, N vs. S, d.f. = 1) and clones (nested within geo, d.f. = 2) on expression (E):

E = T + geo + clone(geo) + T*geo + T*clone(geo) + e;

clone(geo) and T*clone(geo) were treated as random effects. T effect and T*geo interaction were tested against the T*clone(geo) interaction term. Whenever the T*clone(geo) interaction did not approach significance (P > 0.2) the mean squares (MS) associated with it were pooled together with the residual MS and the T effect was tested against the pooled MS [47].

To test for the differential expression present within the N and S clones separately, the MS associated with the T effect were tested against the T*clone interaction within each geographic region. As before, non-significant T*clone MS were pooled with the error MS. Alternatively, paired t-tests were used to compare expression levels in both clones at one temperature to those at the other (equivalent to pooling both the clone MS and the interaction MS with the error MS). The results were remarkably similar; t-tests results are not reported, as they are less conservative. In all cases the results were screened by the False Discovery Rate correction for multiple testing [33].

Lists of differentially expressed genes, in the form of their clusters of orthologous groups (KOG) IDs [34] were submitted to the iPATH web server [48] to generate maps of metabolic and regulatory pathways affected by the differential expression. Fisher’s exact test was used to determine whether a particular pathway was overrepresented among the differentially expressed genes (with the number of non-differentially expressed genes implicated in the given pathway, among total number of non-differentially expressed genes in the dataset as the reference).

All analyses were conducted using JMP 9.0 and JMP Genomics 3.0 (SAS Institute, Gary, NC, USA).

### Availability of supporting data

The data set(s) supporting the results of this article are available as the Additional file 1 included with the article (the list of genes with at least one test significant (FDR < 0.05), including expression data and annotations); expression data are available at GEO (http://www.ncbi.nlm.nih.gov/geo; Accession number GSE53692); gene annotations can be downloaded from http://wfleabase.org.

## Authors’ information

LYY, MEP, and JKC are faculty at, respectively, ETSU, U. of Note Dame and U. of Birmingham; JL is a Laboratory Program Manager at ND, EZ was a Managing Director of the Genomics & Bioinformatics Core Facility at ND and is currently faculty at the University of South Dakota; PJW and KBD were, at the time the study was conducted, a master and an undergraduate student, respectively, at ETSU; currently they are, respectively, a lecturer at Lenoir-Rhyne University and a medical student at ETSU Quillen College of Medicine.

## Electronic supplementary material

Additional file 1: **Expression data and genomic annotation for genes with at least one significant test.** Complete data in http://www.ncbi.nlm.nih.gov/geo/query/acc.cgi?acc=GSE53692. Columns: 1 - Gene ID; 2-25 - expression data (log2 transformed); 26-44 - genomic annotation; 45-57 Data analysis (See Header tab for details). (XLS 2 MB)

## Abbreviations

GxE: Genotype by environment
KOG: (Eukaryotic) clusters of Orthologous Groups
FDR: False Discovery Rate
N: North
S: South
PC: Principal component
E: Expression level
T: Temperature
Geo: Geographic origin
E: Error
MS: Mean squeares
HSP: Heat shock proteins.
